# Hydrothermal Synthesis of Cadmium Sulfide Photocatalyst for Detoxification of Azo Dyes and Ofloxacin Antibiotic in Wastewater

**DOI:** 10.3390/molecules27227944

**Published:** 2022-11-16

**Authors:** Teeradech Senasu, Nattakarn Ruengchai, Sarawoot Khamdon, Narubeth Lorwanishpaisarn, Suwat Nanan

**Affiliations:** 1 Materials Chemistry Research Center, Department of Chemistry and Center of Excellence for Innovation in Chemistry (PERCH-CIC), Faculty of Science, Khon Kaen University, Khon Kaen 40002, Thailand; 2 Department of Biotechnology, Faculty of Technology, Khon Kaen University, Khon Kaen 40002, Thailand

**Keywords:** CdS, degradation, ofloxacin antibiotics, solar light, azo dyes

## Abstract

The complete detoxification of harmful dyes and antibiotics from aqueous solution is essential for environmental remediation. The present work focuses on a facile hydrothermal synthesis of a cadmium sulfide (CdS) photocatalyst using thioacetamide as a sulfur source. The synthesized CdS showed a hexagonal phase with an energy gap of 2.27 eV, suggesting the promising visible-light-responsive semiconducting photocatalyst. The photoactivity of the prepared CdS was investigated by evaluating the degradation of the Reactive red 141 (RR141) dye, Congo red (CR) dye, and ofloxacin (OFL) antibiotic. After only 180 min of solar light illumination, a high performance of 98%, 97%, and 87% toward degradation of RR141, CR, and OFL was obtained. The photodegradation of the pollutants agrees well with the first-order kinetic model. The rate constant of 0.055 min^−1^, 0.040 min^−1^, and 0.026 min^−1^, respectively, was reported toward degradation of RR141, CR, and OFL. Photogenerated holes and hydroxyl radicals play a vital role in removing toxic organic contaminants. The chemical stability of the prepared CdS was also confirmed. The synthesized CdS photocatalyst still maintains high photocatalytic performance even after five consecutive cycles of use, indicating its excellent cycling ability. The present research shows a facile route to fabricate a CdS photocatalyst to completely detoxify harmful organic pollutants, including dyes and antibiotics, in the environment.

## 1. Introduction

Water pollution has become increasingly serious globally. As we know, the quality of freshwater considerably influences human life. Therefore, it is urgent to control the quality of the water. Recently, considerable attention has been paid to the removal of toxic organic pollutants, including dyes and antibiotics contaminated in natural water resources [1,2,3,4,5,6,7,8,9,10,11,12]. Fluoroquinolone antibiotic has been used for the treatment of bacterial-infectious diseases [13,14,15]. Apart from antibiotics, upon industrial development, a massive number of azo dyes have been discharged into natural water. Some dyes are found to be carcinogenic and mutagenic. It is well known that the use of dyes and antibiotics on a large scale causes a serious threat to the environment. Therefore, the complete removal of dyes and antibiotics from the ecosystem is required.

Numerous conventional treatment methods, including adsorption, have been utilized for the incomplete removal of pollutants, with a drawback regarding the generation of secondary pollutants [16]. Alternatively, the photocatalytic method is an effective and environmentally-friendly technique for completely detoxifying harmful contaminants [2,17,18,19]. Generally, the commercially available TiO_2_ photocatalyst is active mostly under UV light. It is generally known that sunlight is composed of 43% visible light and only 5% UV light. Therefore, in terms of energy utilization, the preparation of sunlight-driven photocatalysts have been received considerable attention owing to the benefit of using the economic natural sunlight [5,8].

Visible-light-responsive photocatalysts based on cadmium sulfide have been studied [2,10,16,17,18]. This photocatalyst has been extensively used for the removal of harmful organic contaminants [2,10,16,17,18]. Numerous techniques were used for the preparation of CdS nanostructures [17,20,21,22,23,24]. It is widely known that the hydrothermal technique gives the advantages of simplicity, inexpensive, excellent yield, and promising potential for large-scale fabrication [17,25,26].

The present research focuses on a one-pot synthesis of CdS without the addition of a surfactant, organic solvent, or capping agent. The photocatalytic activity of the prepared catalyst was studied by monitoring the degradation of RR141, CR, and OFL pollutants. The remarkable enhancement of visible-light-responsive performance by up to 94% was obtained. Interestingly, a high photoactivity of 97% was achieved after sunlight irradiation for 100 min. An enhanced performance indicates the excellent environmental remediation property of the prepared CdS for detoxification of harmful dyes and antibiotics contaminated in the environment.

## 2. Experiment

### 2.1. Chemicals

All chemicals were used as received. The ultrapure water (DI, 18.2 MΩ cm) was utilized.

### 2.2. Synthesis of CdS Photocatalyst

The CdS photocatalyst was prepared by a facile hydrothermal method [17]. The Cd^2+^ solution was prepared first by dissolving 3.0847 g of Cd(NO_3_)_3_⋅5H_2_O in 30 mL of DI water. After that, 5.0 M NaOH solution was added until the pH of the solution reached 12. The Cd^2+^ solution with a pH of 12 was denoted as solution A. Separately, the S^2−^ solution was prepared by dissolving 2.2540 g of thioacetamide (TAA) in 30 mL of DI water (denoted as solution B). After solution B was added to solution A, the yellow color and the precipitation were observed. The reaction mixture was then transferred into a 100 mL Teflon-lined autoclave. The temperature was maintained at 120 °C for 12 h. After cooling down to room temperature, the precipitate was collected, washed with water and ethanol, and then dried at 60 °C for 6 h.

### 2.3. Characterization

The characterization of the sample was reported previously [18,27,28]. The chemical state and electronic structure of the prepared CdS were elucidated by X-ray photoelectron spectroscopy (XPS) at BL5.3, SLRI, Nakhon Ratchasima, Thailand. A ULVA-PHI 500 VersaProbe II with monochromatic Al Kα radiation was utilized as an excitation source. The C 1S peak at 284.6 eV was marked as a reference peak for calibration of the binding energy.

### 2.4. Photocatalytic Degradation of the Toxic Pollutants

The photoactivity of the prepared CdS photocatalyst was determined by investigating the removal of RR141, CR, and OFL under visible light (a Panasonic daylight lamp, 15 W) and natural sunlight. The details of the photodegradation study can be found elsewhere [18].

The blank experiment was carried out by irradiating the pollutant solution without the incorporation of the CdS photocatalyst. The photocatalytic degradation study was carried out in 10 ppm aqueous solution of each pollutant (volume of 200 cm^3^). About 50 mg of the CdS photocatalyst was added. The solution of 5 cm^3^ was sampled after light illumination. The concentration of RR141, CR, and OFL was determined by elucidating the absorbance at the maximum wavelength (λ_max_) of 544 nm, 500 nm, and 286 nm, respectively, using UV-vis spectrophotometric method.

The photoactivity toward removal of the pollutant was calculated by Equation (1):

Photoactivity (%) = (1 − C/C_0_) × 100% (1)

where C_0_, and C represent the initial concentration and the concentration of the pollutant solution after a specific time of photo illumination, respectively.

The performance of CdS can also be determined from the degradation rate as follows.

dC/dt = −k_app_C (2)

ln(C_0_/C) = k_app_t (3)

where k_app_ is the apparent first-order rate constant of the degradation reaction.

To investigate the main species involved in the complete removal of the pollutant, t-butanol, NaN_3_, EDTA-2Na, and K_2_Cr_2_O_7_ were added as a quencher of the hydroxyl radicals, superoxide anion radicals, holes, and electrons, respectively. Furthermore, KI was also added for quenching of surface hydroxyl radicals and photogenerated holes. In practice, 5 mM of each scavenger was added [2]. 

To study the reusability of the prepared CdS, after the first cycle, the used CdS catalyst was filtered and washed with ethanol and water [2,18]. This catalyst was then dried before use in the next cycle. The cycling ability was investigated for five successive runs.

## 3. Discussion

### 3.1. Characterization of the CdS Catalyst

**Figure 1:**
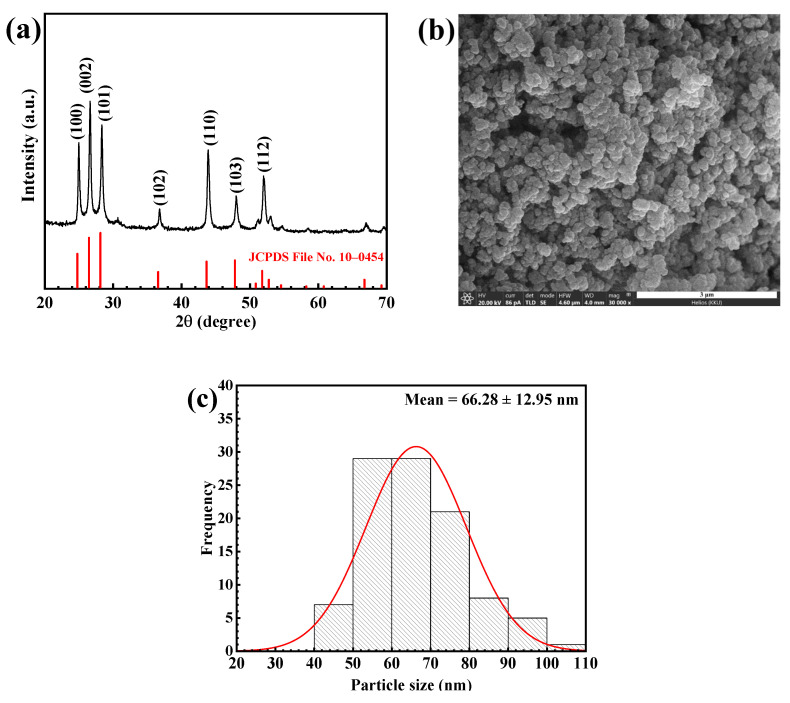
XRD pattern (**a**), FE—SEM micrograph (**b**), and size distribution (**c**) of the CdS nanoparticles.

The XRD pattern of the prepared CdS photocatalyst (Figure 1a) belongs to the hexagonal phase with the diffraction peaks at the 2θ of 25.07°, 26.54°, 28.16°, 36.76°, 43.96°, 48.03° and 51.98° due to the diffraction from the (100), (002), (101), (102), (110), (103) and (112) reflection planes, respectively. The result agrees well with that reported in the JCPDS No. 41-1049 file [6]. The strong peaks demonstrate a well-crystallized structure. The highest intensity of the (002) peak suggests that nanospheres possess an orientation along the (001) direction [29]. The crystallite size, calculated using the Scherrer equation, was found to be 26.76 nm. 

The scanning electron microscopic (SEM) method was used to study morphology and the shape of the synthesized photocatalyst. The Image J analysis was used to calculate the average diameter of the CdS nanomaterials. Figure 1b shows the SEM micrograph of CdS with a spherical morphology of about 66 nm. In addition, the size distribution of the prepared CdS was shown as a histogram in Figure 1c. The results from both XRD and SEM do confirm the chemical structure and the purity of the prepared CdS photocatalyst.

**Figure 2:**
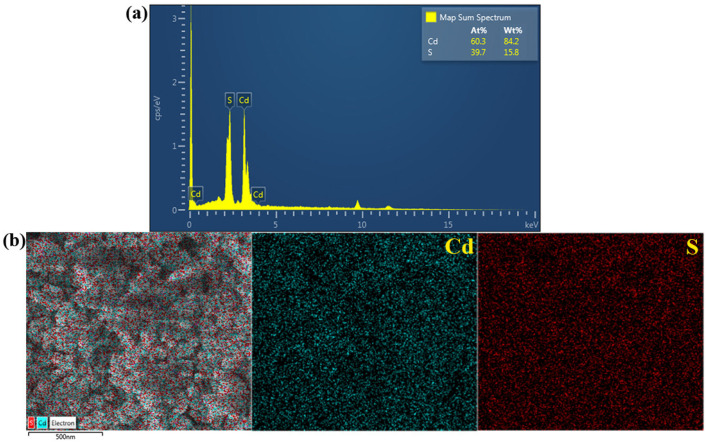
EDX spectrum (**a**), SEM image of the mapping area, and EDX elementary mapping of the Cd and S of CdS nanoparticles (**b**).

The EDX method was used to confirm the elemental composition of the prepared CdS. The EDX spectrum (Figure 2a) suggests the existence of cadmium (Cd) and sulfur (S) elements. The weight% of Cd and S is 84.2% and 15.15.8%, respectively. Accordingly, the atomic% of these elements is 60.3% and 39.7%, respectively. The mapping investigation was also included. The SEM image of the mapping area is presented in Figure 2b. In addition, the elemental color mapping of the sample displayed well dispersion of Cd and S throughout the prepared catalyst indicating the high purity of the synthesized CdS.

The growth mechanism regarding the formation of spherical CdS, based on using thiourea as a sulfur source, was proposed previously in the literature [17]. In the first step, after the addition of the S^2−^ solution to the Cd^2+^ solution, thiourea (TU) acts as a ligand. The stable Cd-TU complex can be generated. After that, during hydrothermal synthesis, the temperature of the system increases to more than 100 °C. The weakening of the Cd-TU complex occurred. This causes the slow release of Cd^2+^ ions. After that, TU can be attacked by oxygen (O) atoms from water (strong nucleophiles). This leads to the weakening of S=C double bonds so that slow release of S^2−^ anions can be found. The formation of CdS nuclei can occur after S^2-^ reacts with the pre-released Cd^2+^. These nuclei will act as seeds for the subsequent crystal growth process. All in all, after the nucleation process, the formation of spherical CdS is expected. The mechanism can be adapted when using thioacetamide (TAA) as a sulfur source. The complex between the Cd^2+^ and TAA can be proposed. Accordingly, the formation of CdS is similar to that explained previously.

**Figure 3:**
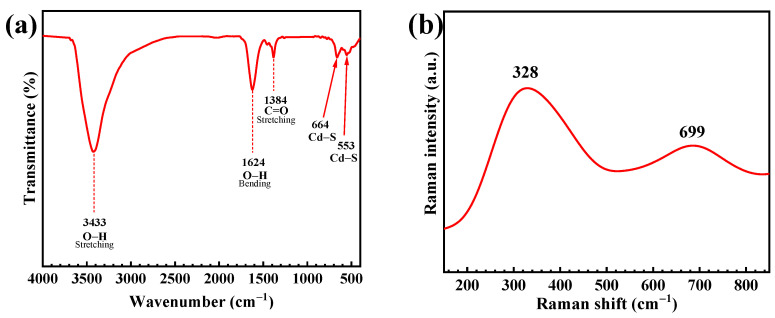

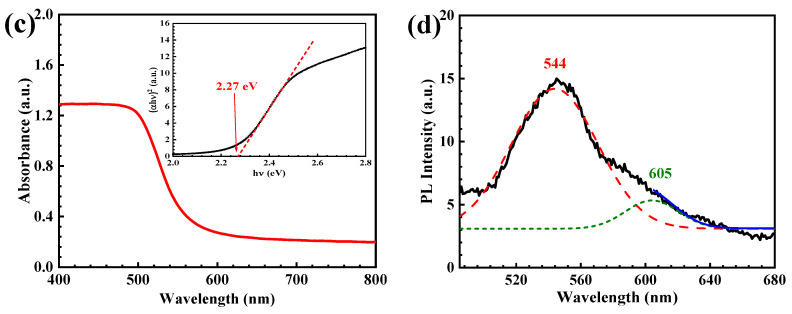
FT−IR spectrum (**a**), Raman spectrum (**b**), diffused reflectance spectrum (**c**) with a Tauc plot for determination of energy band gap (inset plot) and PL spectrum (**d**) of the CdS nanoparticles using an excitation wavelength of 355 nm.

The FT-IR spectrum in Figure 3a exhibited the vibrational bands at 3433 cm^−1^ and 1624 cm^−1^, indicating the O-H stretching and bending vibration of adsorbed water on the CdS surface [17,30]. The band at 1384 cm^−1^ is due to the C=O stretching. Two peaks at 664 cm^−1^ and 553 cm^−1^ are related to the presence of the Cd-S bond [18,31]. The Raman spectrum (Figure 3b) showed two peaks located at 328 cm^−1^ and 699 cm^−1^. These are assigned to the first-order and the second-order longitudinal optical (LO) phonon mode, respectively, found in the prepared CdS.

Figure 3c shows the UV–vis diffuse reflectance spectrum of the prepared CdS with the band energy (Eg) of 2.27 eV, determined from the Tauc plot [18]. Accordingly, the absorption edge of 546 nm over the visible light was obtained. Furthermore, the electron-hole recombination rate of the sample was determined from the photoluminescence spectrum (PL) in Figure 3d. Two peaks located at about 544 nm and 605 nm correspond to the near band edge (NBE) emission and the trapped emission, respectively [17,18,32]. 

**Figure 4:**
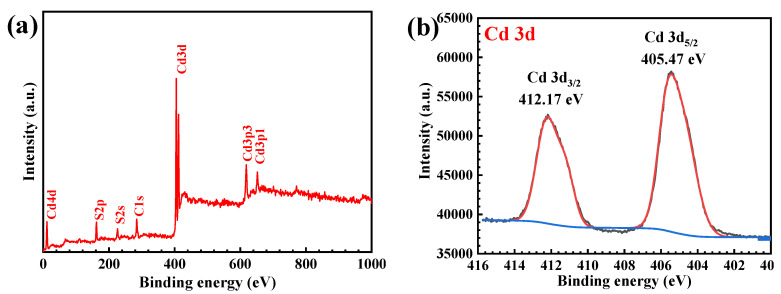

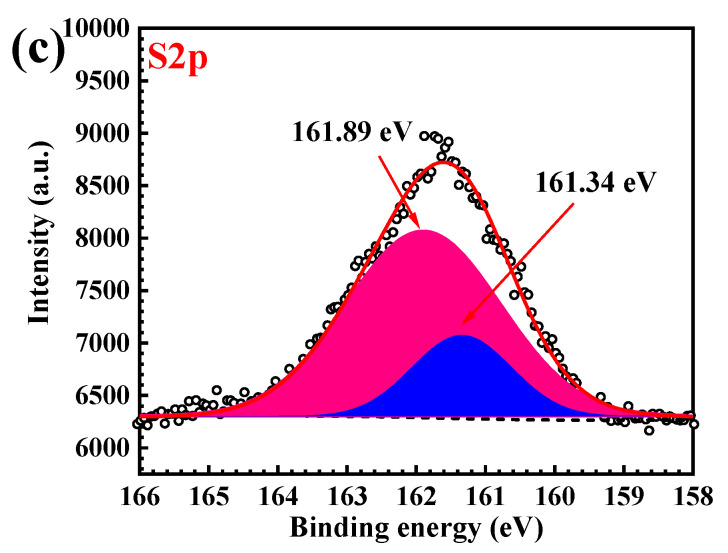
Typical XPS survey scan of CdS (**a**), the high-resolution XPS spectrum of Cd3d (**b**), and S2p (**c**).

The chemical compositions and the chemical state on the surface of the prepared CdS was examined by X-ray photoelectron spectroscopy (XPS). The survey scan of the XPS spectrum (Figure 4a) clearly confirmed the presence of Cd and S elements in the photocatalyst. The high-resolution XPS spectrum of the Cd 3d is displayed in Figure 4b. The two main peaks at 405.47 eV and 412.17 eV are attributed to the contribution of the Cd 3d_5/2_ and Cd 3d_3/2_, respectively. This confirms the existence of Cd^2+^ species in the prepared CdS photocatalyst [14,18,30]. On examining the sulfur element, the XPS spectrum of S 2p (Figure 4c) showed two peaks at 161.34 and 161.89 eV resulting from the existence of S^2−^ from the CdS [14,18].

**Figure 5:**
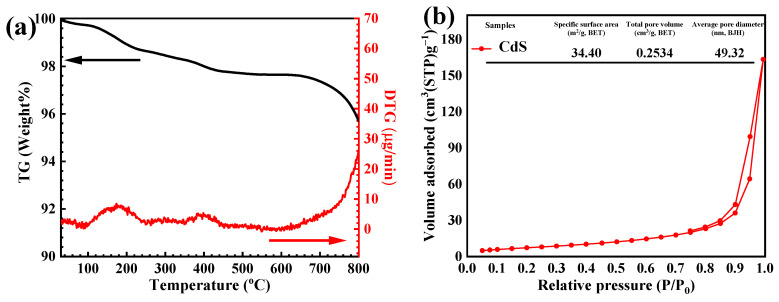

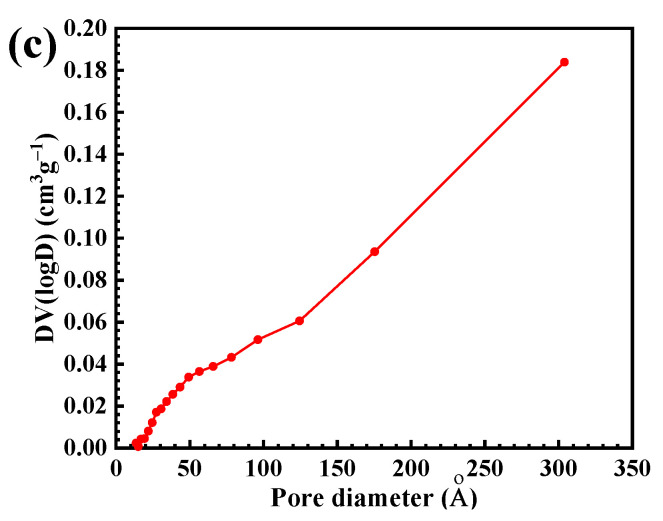
TG thermogram (**a**), N_2_ adsorption−desorption isotherm (**b**), and pore size distribution curve (**c**) of the CdS.

The TG and DTG curves of the prepared CdS (Figure 5a) showed two steps of weight loss over the temperature range of 30–800 °C. The first weight loss of about 1.8% below 200 °C might be assigned to the loss of physically absorbed water molecules [17]. The second weight loss of 4.1% between 300 to 800 °C may be due to the oxidation of cadmium ions in the air atmosphere [17]. The textural properties, including the specific surface area and pore size distribution of CdS, were elucidated from a multipoint BET of the nitrogen (N_2_) adsorption-desorption isotherm. Based on the IUPAC classification, the CdS exhibited a type IV isotherm. In addition, a distinct H_3_ hysteresis loop was found at high relative pressure (Figure 5b) [18]. The pore size distribution of the sample is shown in Figure 5c. A mesoporous catalyst showed a specific surface area of 34 m^2^/g with an average pore volume of 0.25 cm^3^/g and a mean pore diameter of 49 nm.

### 3.2. Photodegradation Study

The removal of RR141, CR dyes, and OFL antibiotic was determined under visible light (a Panasonic daylight lamp, 15 W) and natural sunlight.

#### 3.2.1. Photodegradation of Pollutants

**Figure 6:**
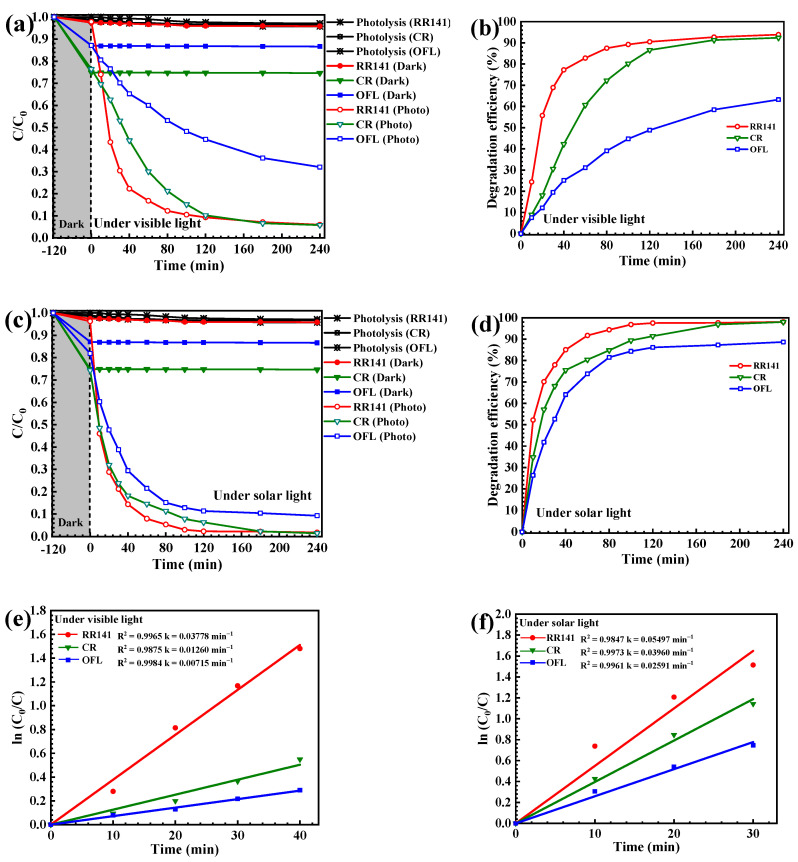
Lowering of the RR141, CR dyes, and OFL antibiotic concentration in the presence of CdS nanoparticles under visible light irradiation (**a**) and solar light irradiation (**c**). Photodegradation efficiency of CdS nanoparticles toward photodegradation of RR141, CR dyes, and OFL antibiotic under visible irradiation (**b**) and solar light irradiation (**d**). A linear plot of ln(C_0_/C) vs. irradiation time toward photodegradation of RR141, CR dyes, and OFL antibiotic under visible light irradiation (**e**) and solar light irradiation (**f**).

As clearly detected in Figure 6a, the lowering of the concentration with time confirmed the removal of all pollutants under visible light. The photolysis of the pollutant is negligible. In the case of RR141, about a 6% removal of RR141 via the adsorption process was detected. Interestingly, nearly a 94% degradation of RR141 was observed under visible light. The adsorption of Congo red (CR) dye by the CdS was less than 30%. The photocatalytic performance of about 93% and 63% was observed toward degradation of CR dye and OFL antibiotic, respectively (Figure 6b). Interestingly, under sunlight, a rapid lowering of pollutant concentration with time was observed (Figure 6c). The CdS photocatalyst showed a 98% and 88% performance toward degradation of the azo dye and the antibiotic, respectively (Figure 6d). The photodegradation reaction follows the first-order reaction (Figure 6e,f) [2,18,33]. In the case of RR141, the corresponding rate constants (k) of 0.013 and 0.055 min^−1^ were reported from the visible light and natural sunlight illumination, respectively. It should be noted that the sunlight photocatalytic performance is greater than that obtained after visible light indicating the real-scale application of the CdS catalyst by utilization of the abundant sunlight. The degradation of toxic contaminants can be carried out practically by economical natural solar energy.

#### 3.2.2. Photocatalytic Degradation Mechanism and Cycling Ability

**Figure 7:**
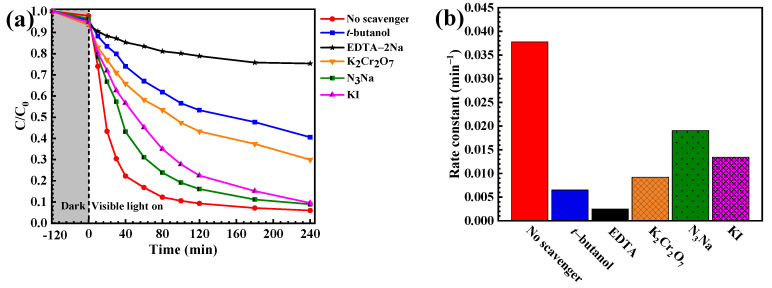
Lowering of the RR141 concentration in the presence of CdS nanoparticles under visible light irradiation (**a**) and rate constant (k) of photodegradation (**b**) in the presence of various scavengers.

The photocatalytic degradation mechanism of toxic pollutants was investigated from the trapping experiment [2]. The effect of some scavengers on the degradation of the pollutant was examined. A sharp lowering of photoactivity was observed after the incorporation of EDTA-2Na and t-butanol (Figure 7a), implying the major role of the photogenerated hole and hydroxyl radicals in the degradation of the pollutant. The rate constant obtained after the addition of the hydroxyl radical scavenger is 5.8 times lower than that detected from the control experiment (no scavenger process). In the case of OFL, our previous reports showed that photogenerated holes are also the main active species involved in the removal of OFL antibiotics [2].

After photo-irradiation, the electrons and holes can be photogenerated in the conduction band (CB) and valence band (VB), respectively. After that, the formation of reactive species then occurred. The CB and VB levels of the CdS were calculated using the Milliken electronegativity theory [18] as shown:

E_VB_ = *χ* − E_C_ + 0.5E_g_ (4)

E_CB_ = E_VB_ − E_g_ (5)

where E_VB_, E_CB_, and E_C_ are the VB, the CB, and the standard hydrogen electrode potential (≈4.5 eV), respectively. *χ* is the absolute value of the electronegativity of the CdS catalyst. The VB and the CB levels of the CdS catalyst are 1.89 and −0.38 eV, respectively. The band gap of the CdS catalyst is 2.27 eV. In summary, the photodegradation mechanism of the toxic contaminant, in the presence of CdS photocatalyst, can be proposed as follows

CdS + hν → CdS + e^−^ + h^+^ (6)

e^−^ + O_2_ → •O_2_^−^ (7)

•O_2_^−^ + 2H_2_O + e^−^ → 2•OH + 2OH^−^ (8)

h^+^ + OH^−^ → •OH (9)

•OH + contaminant → products(10)

h^+^ + contaminant → products (11)

**Figure 8:**
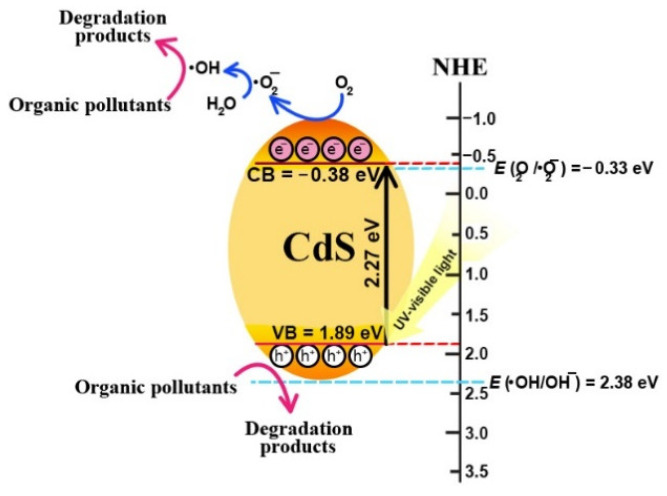
Photocatalytic mechanism schemes of CdS nanoparticles toward the degradation of organic pollutants under UV-visible light irradiation.

The detail of the photocatalytic degradation mechanism is summarized in Figure 8.

For better understanding, the pathway of RR141 dye degradation was investigated previously in our group based on the results from the LC-MS technique [3]. The mass spectrum obtained from the photodegradation intermediate products of RR141 dye was identified. The degradation mechanism was also proposed using the existence of some important breakdown products [3]. In addition, the LC-MS was also used to propose the photocatalytic degradation pathway of the OFL antibiotic [2].

**Figure 9:**
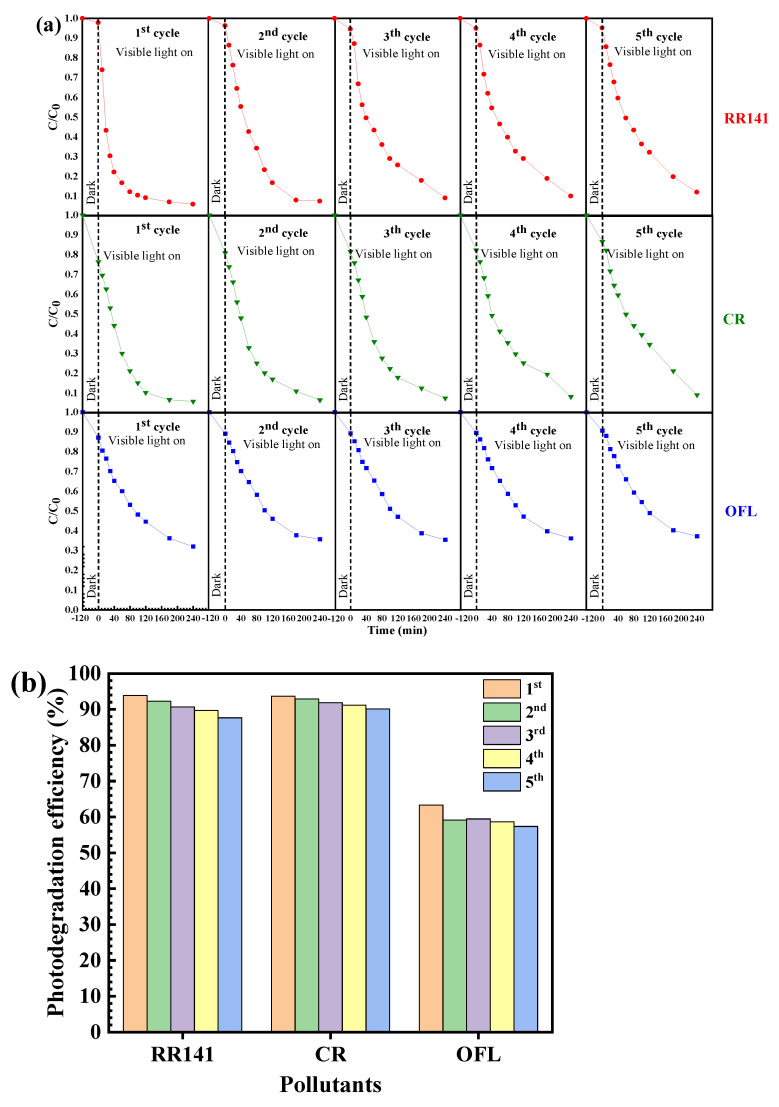
Reusability of the CdS nanoparticles for photodegradation of the RR141, CR dyes, and OFL antibiotic for five cycles under visible light irradiation (**a**), bar chart (**b**) showing the photocatalytic performance of the CdS nanoparticles toward photodegradation of the RR141, CR dyes, and OFL antibiotic for five cycles.

**Figure 10:**
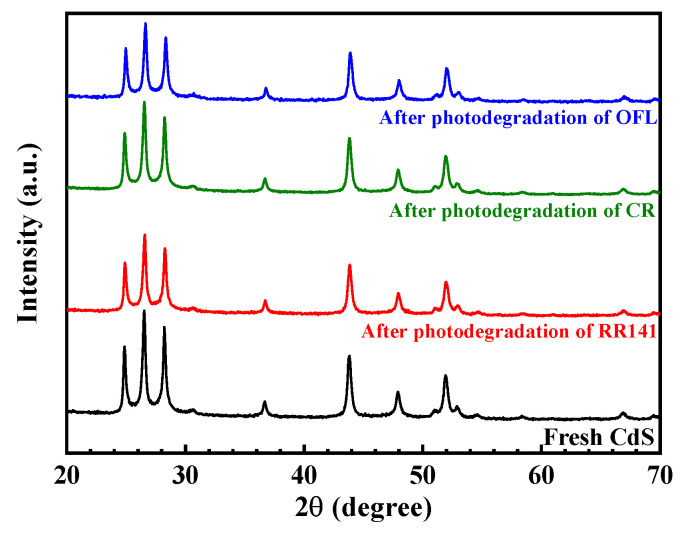
XRD patterns of the CdS nanoparticles before and after photodegradation of RR141, CR dyes, and OFL antibiotic.

Reusability is a major factor influencing the practical use of the catalyst [2,18]. Therefore, the reuse of the prepared CdS after the degradation of the pollutants was investigated. The prepared CdS catalyst still shows great performance even after five times of use (Figure 9). The chemical structure of the CdS after the removal of the pollutants was also elucidated. The XRD patterns of the used and the fresh CdS (Figure 10) are similar, confirming the prepared photocatalyst’s structural stability. 

It is also important to note that Cd metal is toxic. The application of the CdS photocatalyst is based on its advantage of high photocatalytic performance under sunlight. However, the stability of the CdS photocatalyst has to be taken into consideration. The possibility of photo-corrosion, found in the CdS photocatalyst toward photodegradation of the harmful organic pollutants, was worth future work. In our previous work, the concentration of Cd^2+^ in the pollutant solution was investigated [7]. However, the amount of Cd^2+^ was quite low. The improvement could be achieved by synthesizing the photocatalyst with an anti-photo-corrosion property. This is suggested for further work.

The photocatalytic performance of the various photocatalysts toward the removal of dyes and antibiotics has been studied previously [2,4,5,6,7,25,34,35]. In this work, the prepared CdS photocatalyst is used for the degradation of RR141 dye, CR dye, and OFL antibiotic under visible light irradiation. The photocatalytic performance of the synthesized CdS compared to those shown in the previous works is tabulated in Table 1. On examining RR141 degradation, the bare ZnO showed an efficiency of 95–98% [1,2], while the SDS-capped ZnO exhibited 60% and 95% photoactivity under visible light and UV light, respectively [3]. The metal-doped ZnO performed from 89 to 96% [36,37]. The ZnO/CdS composite showed a high performance of 80% within 120 min. In the case of bare bismuth molybdate photocatalyst, an efficiency of 30–70% was obtained [4,5]. Interestingly, the CdS in the present work provided a high performance of 93–98% under visible light and natural sunlight. On examining CR dye removal, the bare CdS from the previous results showed a photoactivity of 31–91% [6,7,8,38]. The composites based on CdS enhanced the performance by 82–95% [8,38,39]. It should be noted that the CdS in the present work displayed high efficiency of 91–97 without the creation of heterojunction. In terms of OFL degradation, the pristine CdS from the previous works showed an efficiency of 70–79% [2,9]. The CdS-based binary nanocomposites displayed a photoactivity of 61–86% [2,10,11]. Interestingly, high sunlight performance of 89% was obtained from the CdS in the present work.

In this research, the prepared CdS photocatalyst provided high sunlight performance of 98% and 88% toward degradation of dye and antibiotic, respectively, without doping the noble metals or creating the heterostructures. The present research demonstrates how to prepare the novel photocatalyst for completely detoxifying the harmful contaminants in natural water by applying natural sunlight.

## 4. Conclusions

This work reports a facile hydrothermal synthesis of CdS semiconducting photocatalysts without the addition of surfactant, organic solvent, or capping agent. The hexagonal CdS showed a band energy of 2.27 eV. The performance of 98% and 88% under sunlight was achieved toward degradation of the azo dye and ofloxacin antibiotic, respectively. The photodegradation of the pollutant agrees well with the first-order reaction. Hydroxyl radicals play a crucial role in the removal of pollutants. The prepared CdS photocatalyst still shows promising efficiency after the fifth cycle suggesting the great reusability of the sample. The present finding offers a novel route to create a sunlight-active CdS photocatalyst for environmental protection.

## Figures and Tables

**Table 1:** Comparison of dyes and antibiotic degradation by using various photocatalysts.

Catalyst	Conc.	Catalyst Loading	Light Source	Lamp	Time (min)	Degradation (%)	Ref.
**Photodegradation of Reactive Red 141 (RR141) azo dye**							
ZnO	10 mgL^−1^	50 mg	UV	125 W Hg lamp	240	95	[1]
ZnO	10 mgL^−1^	50 mg	UV	125 W Hg lamp	240	98	[2]
SDS capped ZnO	10 mgL^−1^	50 mg	UV	125 W Hg lamp	240	95	[3]
SDS capped ZnO	10 mgL^−1^	50 mg	Visible	15 W	240	60	[3]
Cu-ZnO	50 mgL^−1^	100 mg	UV	-	120	89	[36]
3% Pb-ZnO	30 mgL^−1^	30 mg	UV	-	120	96	[37]
ZnO/CdS	10 mgL^−1^	50 mg	Visible	15 W	120	80	[2]
Bi_2_MoO_6_	10 mgL^−1^	50 mg	UV	125 W Hg lamp	240	37	[4]
Bi_2_MoO_6_	10 mgL^−1^	50 mg	Visible	15 W	240	45	[4]
Bi_4_MoO_9_	10 mgL^−1^	50 mg	UV	125 W Hg lamp	240	68	[5]
Bi_4_MoO_9_	10 mgL^−1^	50 mg	Sunlight	-	240	70	[5]
CdS	10 mgL^−1^	50 mg	Visible	15 W	240	93	This work
CdS	10 mgL^−1^	50 mg	Sunlight	-	240	98	This work
**Photodegradation of Congo Red (CR) dye**							
CdS	30 mgL^−1^	30 mg	Visible	300 W xenon lamp	80	90	[6]
CdS	10 mgL^−1^	50 mg	Visible	800 W xenon lamp	60	91	[7]
CdS	10 mgL^−1^	250 mg	Sunlight	-	120	31	[38]
CdS	25 mgL^−1^	15 mg	Sunlight	-	300	85	[8]
TiO_2_-CdS	25 mgL^−1^	15 mg	Sunlight	-	300	95	[8]
ZnO-CdS	10 mgL^−1^	-	UV	250 W Hg lamp	100	88	[39]
CdS&NiO/Ni_2_O_3_	10 mgL^−1^	250 mg	Sunlight	-	120	82	[38]
CdS	10 mgL^−1^	50 mg	Visible	15 W	180	91	This work
CdS	10 mgL^−1^	50 mg	Sunlight	-	180	97	This work
**Photodegradation of Ofloxacin (OFL) antibiotic**							
CdS	10 mgL^−1^	50 mg	Visible	15 W	240	70	[2]
CdS	10 mgL^−1^	25 mg	Visible	85 W	80	79	[9]
ZnO/CdS	10 mgL^−1^	50 mg	Visible	15 W	240	73	[2]
CdS/MoS_2_	10 mgL^−1^	100 mg	Visible	400 W xenon lamp	90	61	[10]
CdS/TiO_2_	10 mgL^−1^	450 mg	Visible	85 W	180	86	[11]
CdS	10 mgL^−1^	50 mg	Visible	15 W	240	63	This work
CdS	10 mgL^−1^	50 mg	Sunlight	-	240	89	This work

## Data Availability

Not applicable.

## Sample Availability

Samples of the compounds are not available from the authors.
